# Supplementary material for: Mechanisms governing the pioneering and redistribution capabilities of the non-classical pioneer PU.1
Source: Nat Commun. 2020 Jan 21;11:402. doi: 10.1038/s41467-019-13960-2 (PMC6972792; doi:10.1038/s41467-019-13960-2)
Supplement: Supplementary file 7 — Source data [file 41467_2019_13960_MOESM7_ESM.zip › Source_Data/Figure6/Figure6B_MotifScanOutput/ETS1_PU.1/homerResults.html]

/misc/data/analysis/project\_PU1/CTV1/motifs/basic/peaksETS1\_DSG\_PU1\_merged/final - Homer de novo Motif Results


# Homer *de novo* Motif Results (/misc/data/analysis/project\_PU1/CTV1/motifs/basic/peaksETS1\_DSG\_PU1\_merged/final)

Known Motif Enrichment Results  
Gene Ontology Enrichment Results  
If Homer is having trouble matching a motif to a known motif, try copy/pasting the matrix file into
STAMP  
More information on motif finding results: HOMER
| Description of Results
| Tips
  
Total target sequences = 15830  
Total background sequences = 32072  
\* - possible false positive  

|  |  |  |  |  |  |  |  |  |
| --- | --- | --- | --- | --- | --- | --- | --- | --- |
| Rank | Motif | P-value | log P-pvalue | % of Targets | % of Background | STD(Bg STD) | Best Match/Details | Motif File |
| 1 | C T G A C T G A T A G C T G C A C T A G A C T G C G T A C G T A T C A G G A C T T C A G | 1e-3181 | -7.325e+03 | 79.08% | 22.21% | 44.9bp (71.9bp) | Etv2(ETS)/ES-ER71-ChIP-Seq(GSE59402)/Homer(0.967)(0.966) More Information \| Similar Motifs Found | motif file (matrix) |
| 2 | C T A G T G A C G T C A C T A G T C A G G T C A C G A T T A C G A G C T A C T G A C T G A C G T A G C T C G A T | 1e-978 | -2.252e+03 | 35.98% | 9.94% | 45.2bp (60.6bp) | ETS:RUNX(ETS,Runt)/Jurkat-RUNX1-ChIP-Seq(GSE17954)/Homer(0.954) More Information \| Similar Motifs Found | motif file (matrix) |
| 3 | A G T C C G T A A G T C A C G T A C G T A G T C A G C T | 1e-865 | -1.994e+03 | 37.96% | 12.41% | 55.1bp (69.9bp) | PU.1(ETS)/ThioMac-PU.1-ChIP-Seq(GSE21512)/Homer(0.764) More Information \| Similar Motifs Found | motif file (matrix) |
| 4 | A C T G C G A T C G T A T C G A A G T C A G T C C T G A A T G C C G T A | 1e-743 | -1.712e+03 | 33.13% | 10.65% | 51.2bp (62.1bp) | RUNX(Runt)/HPC7-Runx1-ChIP-Seq(GSE22178)/Homer(0.923) More Information \| Similar Motifs Found | motif file (matrix) |
| 5 | A T C G A C G T C G A T G A T C A G T C A G T C A G T C G C A T A G C T G A C T A G C T | 1e-619 | -1.428e+03 | 26.25% | 7.75% | 53.1bp (75.9bp) | SpiB(ETS)/OCILY3-SPIB-ChIP-Seq(GSE56857)/Homer(0.842) More Information \| Similar Motifs Found | motif file (matrix) |
| 6 | A G T C C G T A A C G T A G T C A G T C A C T G A T C G | 1e-395 | -9.096e+02 | 20.94% | 7.19% | 48.6bp (68.4bp) | ETV5/MA0765.1/Jaspar(0.923) More Information \| Similar Motifs Found | motif file (matrix) |
| 7 | C G T A A T G C C G A T C G A T T G A C G A T C A G T C G C A T A C G T G C A T | 1e-248 | -5.712e+02 | 11.69% | 3.50% | 53.1bp (71.0bp) | ELF5(ETS)/T47D-ELF5-ChIP-Seq(GSE30407)/Homer(0.808) More Information \| Similar Motifs Found | motif file (matrix) |
| 8 | G T A C T C A G A T C G A G T C A G T C C T G A G T A C C G T A | 1e-216 | -4.995e+02 | 28.02% | 15.62% | 53.0bp (63.1bp) | MET32/MA0334.1/Jaspar(0.895) More Information \| Similar Motifs Found | motif file (matrix) |
| 9 | C G T A C T G A A G T C G A C T C T A G G A T C G T C A C T G A | 1e-211 | -4.877e+02 | 27.98% | 15.72% | 55.8bp (66.4bp) | AMYB(HTH)/Testes-AMYB-ChIP-Seq(GSE44588)/Homer(0.874) More Information \| Similar Motifs Found | motif file (matrix) |
| 10 | A C G T A G T C A G T C A C G T A G T C A G T C A C T G A G T C | 1e-184 | -4.241e+02 | 28.86% | 17.18% | 56.0bp (73.0bp) | SRSF1(RRM)/Homo\_sapiens-RNCMPT00107-PBM/HughesRNA(0.892) More Information \| Similar Motifs Found | motif file (matrix) |
| 11 | A C G T A T C G A C G T A C G T A G T C A G T C A C T G A G T C | 1e-181 | -4.183e+02 | 14.91% | 6.54% | 55.0bp (71.7bp) | RDR1/MA0360.1/Jaspar(0.879) More Information \| Similar Motifs Found | motif file (matrix) |
| 12 | T G C A A T G C G A C T T C G A G A T C T G C A G C T A A G C T G A C T G A T C A G T C G T A C | 1e-171 | -3.940e+02 | 4.87% | 0.79% | 47.7bp (68.1bp) | GFY(?)/Promoter/Homer(0.965) More Information \| Similar Motifs Found | motif file (matrix) |
| 13 | T A C G A G T C A C T G T A G C C G A T C G T A A T C G | 1e-168 | -3.877e+02 | 26.23% | 15.48% | 55.3bp (71.0bp) | STP3/MA0396.1/Jaspar(0.866) More Information \| Similar Motifs Found | motif file (matrix) |
| 14 | A C T G C G A T T C G A T C G A A T G C G A C T A C G T A G T C G A T C G A T C G A C T G C T A | 1e-153 | -3.532e+02 | 10.92% | 4.36% | 56.3bp (66.7bp) | PB0058.1\_Sfpi1\_1/Jaspar(0.707) More Information \| Similar Motifs Found | motif file (matrix) |
| 15 | G A T C C T G A A T C G C G A T C G A T A T G C A G T C G A C T A G T C G C T A T C G A C G T A | 1e-147 | -3.407e+02 | 8.33% | 2.82% | 53.8bp (67.0bp) | PB0058.1\_Sfpi1\_1/Jaspar(0.790) More Information \| Similar Motifs Found | motif file (matrix) |
| 16 | T C G A A C G T A C T G C G T A T A C G A C G T G T A C C G T A A C G T A G T C | 1e-143 | -3.304e+02 | 8.29% | 2.87% | 52.8bp (60.0bp) | JunB(bZIP)/DendriticCells-Junb-ChIP-Seq(GSE36099)/Homer(0.988) More Information \| Similar Motifs Found | motif file (matrix) |
| 17 | C G T A C G T A A T G C A G T C C T G A A T C G C G T A A C G T | 1e-132 | -3.043e+02 | 7.52% | 2.56% | 57.6bp (62.3bp) | grh/dmmpmm(Bigfoot)/fly(0.785) More Information \| Similar Motifs Found | motif file (matrix) |
| 18 | G A T C T G A C A G T C C A G T A G T C A G T C A G T C G T A C A G T C C G T A A G T C | 1e-127 | -2.927e+02 | 15.73% | 8.31% | 54.0bp (63.7bp) | EGR1/MA0162.2/Jaspar(0.897) More Information \| Similar Motifs Found | motif file (matrix) |
| 19 | A G T C G T C A A C G T A G T C A G T C A C G T A T G C A C G T | 1e-118 | -2.733e+02 | 11.24% | 5.23% | 54.8bp (64.5bp) | Ets1-distal(ETS)/CD4+-PolII-ChIP-Seq(Barski\_et\_al.)/Homer(0.810) More Information \| Similar Motifs Found | motif file (matrix) |
| 20 | C T A G T C A G T C G A C T G A C T A G T C A G T C G A C T G A T C A G C T G A T C A G T C G A T C G A T C A G | 1e-116 | -2.690e+02 | 6.10% | 1.94% | 51.2bp (75.7bp) | PU.1(ETS)/ThioMac-PU.1-ChIP-Seq(GSE21512)/Homer(0.727) More Information \| Similar Motifs Found | motif file (matrix) |
| 21 | T G C A A T G C A G C T C A G T A G T C A G T C A T C G G A T C A G C T A G C T A G T C A T G C | 1e-114 | -2.629e+02 | 7.40% | 2.76% | 52.7bp (75.8bp) | RDR1/MA0360.1/Jaspar(0.829) More Information \| Similar Motifs Found | motif file (matrix) |
| 22 | G T A C T C G A T C G A G A T C G T C A C T A G A G T C G T C A C T A G | 1e-105 | -2.419e+02 | 36.58% | 26.78% | 56.4bp (65.9bp) | MyoG(bHLH)/C2C12-MyoG-ChIP-Seq(GSE36024)/Homer(0.809) More Information \| Similar Motifs Found | motif file (matrix) |
| 23 | C T G A T C G A A G T C A G T C C G T A A G T C C G T A C G A T T A G C C G T A T C G A C G T A T C A G T G C A | 1e-101 | -2.343e+02 | 4.23% | 1.09% | 45.6bp (67.5bp) | RUNX2(Runt)/PCa-RUNX2-ChIP-Seq(GSE33889)/Homer(0.737) More Information \| Similar Motifs Found | motif file (matrix) |
| 24 | A G T C A C G T A C G T C G T A A C G T A G T C C G A T G A T C | 1e-99 | -2.294e+02 | 17.15% | 10.19% | 55.4bp (64.4bp) | GATA3(Zf)/iTreg-Gata3-ChIP-Seq(GSE20898)/Homer(0.959) More Information \| Similar Motifs Found | motif file (matrix) |
| 25 | A G C T C A G T A C G T A T G C G A T C C A T G A T C G G A T C A C T G A T C G T A G C | 1e-75 | -1.748e+02 | 3.67% | 1.09% | 48.3bp (70.8bp) | PDR1/PDR1\_YPD/[](Harbison)/Yeast(0.771) More Information \| Similar Motifs Found | motif file (matrix) |
| 26 | C G T A C T G A A C G T A G T C C T G A A C T G G T A C | 1e-74 | -1.725e+02 | 41.11% | 32.53% | 57.2bp (65.9bp) | MafA(bZIP)/Islet-MafA-ChIP-Seq(GSE30298)/Homer(0.769) More Information \| Similar Motifs Found | motif file (matrix) |
| 27 | C T A G A C T G A C T G G A T C A C T G A C T G C T A G T C A G A G T C | 1e-71 | -1.637e+02 | 9.62% | 5.18% | 57.7bp (65.4bp) | Sp1(Zf)/Promoter/Homer(0.942) More Information \| Similar Motifs Found | motif file (matrix) |
| 28 | C A T G A T G C A C G T A C T G T C G A G A C T C T G A G T C A G A T C C T G A | 1e-61 | -1.427e+02 | 21.60% | 15.38% | 56.9bp (62.2bp) | elt-3/MA0542.1/Jaspar(0.797) More Information \| Similar Motifs Found | motif file (matrix) |
| 29 | A T G C A C G T C A G T C G A T T A C G C G T A C G A T T A C G | 1e-55 | -1.289e+02 | 13.66% | 8.86% | 54.2bp (66.5bp) | TCF7L2/MA0523.1/Jaspar(0.926) More Information \| Similar Motifs Found | motif file (matrix) |
| 30 | A C T G G T C A A T G C G A C T A G T C C T A G A C G T C T G A A C T G | 1e-53 | -1.238e+02 | 2.43% | 0.68% | 48.5bp (67.6bp) | GFY(?)/Promoter/Homer(0.726) More Information \| Similar Motifs Found | motif file (matrix) |
| 31 | C T G A C G T A A G T C G T C A A C T G A C T G C G T A C G T A A G T C | 1e-49 | -1.142e+02 | 2.30% | 0.66% | 52.8bp (70.9bp) | Ets1-distal(ETS)/CD4+-PolII-ChIP-Seq(Barski\_et\_al.)/Homer(0.800) More Information \| Similar Motifs Found | motif file (matrix) |
| 32 | T G A C C T G A A T G C A G T C G C T A A C T G C T A G C A T G C A T G T A C G G A T C T C A G | 1e-47 | -1.086e+02 | 3.94% | 1.70% | 57.3bp (61.3bp) | BORIS(Zf)/K562-CTCFL-ChIP-Seq(GSE32465)/Homer(0.911) More Information \| Similar Motifs Found | motif file (matrix) |
| 33 | G A T C G A C T A C T G T G A C C A T G A T C G A G C T A C G T A C G T G C A T A C T G G T C A | 1e-45 | -1.045e+02 | 2.27% | 0.69% | 52.7bp (66.9bp) | Bgb::run/MA0242.1/Jaspar(0.764) More Information \| Similar Motifs Found | motif file (matrix) |
| 34 | C T A G T G A C T C A G T A G C G C T A C A G T A T C G A T G C A C T G G A T C T C G A | 1e-44 | -1.035e+02 | 2.63% | 0.91% | 55.7bp (67.4bp) | NRF1(NRF)/MCF7-NRF1-ChIP-Seq(Unpublished)/Homer(0.965) More Information \| Similar Motifs Found | motif file (matrix) |
| 35 | T C G A C T G A T A G C G C A T A T C G T C G A T G A C C G T A T C A G G C A T C G A T | 1e-41 | -9.581e+01 | 4.38% | 2.11% | 56.6bp (63.5bp) | JGL(C2H2)/col-JGL-DAP-Seq(GSE60143)/Homer(0.790) More Information \| Similar Motifs Found | motif file (matrix) |
| 36 | C G T A T A C G C T G A A C T G C T A G T G C A C G T A T A C G T C G A C G T A T C A G T G C A C T G A C T A G | 1e-40 | -9.316e+01 | 2.07% | 0.64% | 54.2bp (53.4bp) | SPIB/MA0081.1/Jaspar(0.677) More Information \| Similar Motifs Found | motif file (matrix) |
| 37 | A G C T A T G C C T G A G A T C C T A G A G C T A T C G C T G A A G T C T C A G | 1e-36 | -8.469e+01 | 3.86% | 1.86% | 55.2bp (56.7bp) | FEA4(bZIP)/Corn-FEA4-ChIP-Seq(GSE61954)/Homer(0.919) More Information \| Similar Motifs Found | motif file (matrix) |
| 38 | A C T G A G T C C G T A A C G T A T C G A G T C A C G T | 1e-36 | -8.375e+01 | 8.95% | 5.78% | 55.7bp (66.2bp) | EIF-2ALPHA(S1)/Drosophila\_melanogaster-RNCMPT00273-PBM/HughesRNA(0.856) More Information \| Similar Motifs Found | motif file (matrix) |
| 39 | C G T A C A G T C G A T A C T G A C T G A C T G A G T C A C T G | 1e-35 | -8.223e+01 | 7.58% | 4.70% | 57.0bp (65.4bp) | btd/MA0443.1/Jaspar(0.806) More Information \| Similar Motifs Found | motif file (matrix) |
| 40 | G T C A C T G A T A C G T G C A A T C G A T C G C G T A A C T G A T C G T G C A C T G A A T C G C T G A | 1e-34 | -8.026e+01 | 2.67% | 1.09% | 59.5bp (67.2bp) | SF2(RRM)/Drosophila\_melanogaster-RNCMPT00066-PBM/HughesRNA(0.701) More Information \| Similar Motifs Found | motif file (matrix) |
| 41 | C T G A C G A T C G A T G T A C A G C T A C T G C T G A A C T G C G T A C G T A | 1e-33 | -7.739e+01 | 3.08% | 1.39% | 51.7bp (69.2bp) | STAT5(Stat)/mCD4+-Stat5-ChIP-Seq(GSE12346)/Homer(0.961) More Information \| Similar Motifs Found | motif file (matrix) |
| 42 | T G C A C T G A T C A G C T G A A C G T A C T G A C T G A G T C A C T G T C A G G A T C A T G C A T G C A T G C | 1e-29 | -6.830e+01 | 1.24% | 0.32% | 55.6bp (60.2bp) | YY1(Zf)/Promoter/Homer(0.936) More Information \| Similar Motifs Found | motif file (matrix) |
| 43 | A T C G A T C G G A T C A G C T A C G T A G T C A G T C A C G T A C G T G T A C A G T C A T C G A T G C T G A C | 1e-28 | -6.638e+01 | 3.15% | 1.54% | 54.5bp (70.5bp) | SRSF9(RRM)/Homo\_sapiens-RNCMPT00067-PBM/HughesRNA(0.788) More Information \| Similar Motifs Found | motif file (matrix) |
| 44 | C T A G T C G A G T C A A T C G C T G A G C T A A C T G T G C A G T C A A C T G T C G A A C T G A C T G C T G A | 1e-27 | -6.435e+01 | 0.85% | 0.16% | 56.7bp (51.1bp) | Unknown4/Arabidopsis-Promoters/Homer(0.724) More Information \| Similar Motifs Found | motif file (matrix) |
| 45 | A T G C T C A G T A C G A T G C G T A C G C T A T C G A A G C T T A G C T C A G | 1e-25 | -5.961e+01 | 3.04% | 1.54% | 56.4bp (59.7bp) | NFY(CCAAT)/Promoter/Homer(0.929) More Information \| Similar Motifs Found | motif file (matrix) |
| 46 | G C A T A T C G C G T A A C G T C T G A C T G A T A G C G T C A A G C T A G T C G C T A | 1e-24 | -5.586e+01 | 0.71% | 0.13% | 51.3bp (41.0bp) | GATA(Zf),IR3/iTreg-Gata3-ChIP-Seq(GSE20898)/Homer(0.832) More Information \| Similar Motifs Found | motif file (matrix) |
| 47 | A G C T A G T C A G T C A C T G A G T C A G T C A G T C C G A T | 1e-23 | -5.335e+01 | 3.54% | 1.98% | 56.8bp (64.4bp) | btd/MA0443.1/Jaspar(0.816) More Information \| Similar Motifs Found | motif file (matrix) |
| 48 | A C T G A C T G A C T G A C T G A C G T C G T A A C T G A C G T | 1e-23 | -5.322e+01 | 4.97% | 3.09% | 53.6bp (63.6bp) | Rbm42(RRM)/Xenopus\_tropicalis-RNCMPT00282-PBM/HughesRNA(0.734) More Information \| Similar Motifs Found | motif file (matrix) |
| 49 | T C A G T C G A C G A T G A C T C T G A T A C G C A G T A T G C C G T A | 1e-22 | -5.198e+01 | 2.38% | 1.15% | 55.5bp (62.2bp) | YAP1(MacIsaac)/Yeast(0.804) More Information \| Similar Motifs Found | motif file (matrix) |
| 50 | A G C T A G C T A T C G G A T C A C T G T C A G C G A T T A G C A T G C | 1e-21 | -4.870e+01 | 2.36% | 1.17% | 52.2bp (63.2bp) | CG7903(RRM)/Drosophila\_melanogaster-RNCMPT00144-PBM/HughesRNA(0.779) More Information \| Similar Motifs Found | motif file (matrix) |
| 51 | A C G T A G C T A C T G A C G T C G T A A C T G A C G T | 1e-20 | -4.693e+01 | 3.73% | 2.21% | 52.1bp (65.7bp) | Rbm42(RRM)/Xenopus\_tropicalis-RNCMPT00282-PBM/HughesRNA(0.838) More Information \| Similar Motifs Found | motif file (matrix) |
| 52 | T C G A T C A G G C A T A C G T G A T C G A C T G A T C T C G A A G T C T C A G T C G A A C T G T C G A | 1e-19 | -4.507e+01 | 1.26% | 0.47% | 60.7bp (63.5bp) | ZBTB33(Zf)/GM12878-ZBTB33-ChIP-Seq(GSE32465)/Homer(0.835) More Information \| Similar Motifs Found | motif file (matrix) |
| 53 | A T C G T C G A T A C G A T C G G T A C C T G A A C T G C T A G T G C A C T G A C T G A A C T G C T G A | 1e-19 | -4.473e+01 | 1.89% | 0.88% | 53.7bp (64.0bp) | E2F6/MA0471.1/Jaspar(0.602) More Information \| Similar Motifs Found | motif file (matrix) |
| 54 | T A C G A G T C C T A G C T A G C G T A C T G A C T G A A C G T C G T A T A C G T A G C A T G C C T A G C G T A | 1e-16 | -3.846e+01 | 0.30% | 0.02% | 44.3bp (43.5bp) | YRM1/MA0438.1/Jaspar(0.710) More Information \| Similar Motifs Found | motif file (matrix) |
| 55 | A G C T G A T C A C G T G A C T A G T C C G A T A T G C A G T C G A C T C G A T A G T C A G C T A C G T G A T C | 1e-16 | -3.812e+01 | 0.94% | 0.32% | 55.5bp (70.1bp) | PABPN1(RRM)/Homo\_sapiens-RNCMPT00157-PBM/HughesRNA(0.662) More Information \| Similar Motifs Found | motif file (matrix) |
| 56 | A C T G A G T C A G T C A C T G A G C T G T C A C G T A | 1e-16 | -3.776e+01 | 6.05% | 4.27% | 56.2bp (70.6bp) | At5g08750(C3H)/col-At5g08750-DAP-Seq(GSE60143)/Homer(0.867) More Information \| Similar Motifs Found | motif file (matrix) |
| 57 | A T G C A G T C A G T C A C G T A C G T G T A C A T G C A C G T A G T C A G T C T A G C G T A C | 1e-16 | -3.755e+01 | 1.64% | 0.78% | 50.2bp (64.2bp) | NCU02404(RRM)/Neurospora\_crassa-RNCMPT00238-PBM/HughesRNA(0.746) More Information \| Similar Motifs Found | motif file (matrix) |
| 58 | A G T C A G T C C T A G C T A G T A G C C T G A C G A T C T A G A G T C | 1e-16 | -3.708e+01 | 1.91% | 0.98% | 53.7bp (65.0bp) | ERF6/MA1006.1/Jaspar(0.751) More Information \| Similar Motifs Found | motif file (matrix) |
| 59 | C G T A A C T G A C T G T C G A C G T A T A C G C G T A G C T A T A C G C T G A A C T G C T A G G T C A C G T A | 1e-13 | -3.205e+01 | 0.49% | 0.11% | 47.5bp (0.0bp) | TRA2(RRM)/Drosophila\_melanogaster-RNCMPT00078-PBM/HughesRNA(0.661) More Information \| Similar Motifs Found | motif file (matrix) |
| 60 | A C T G G C T A C T G A C T A G T C G A C T A G C T A G G T C A A C T G T C A G C G T A A C T G C G T A | 1e-12 | -2.956e+01 | 1.40% | 0.69% | 56.2bp (70.3bp) | SRSF1(RRM)/Homo\_sapiens-RNCMPT00163-PBM/HughesRNA(0.721) More Information \| Similar Motifs Found | motif file (matrix) |
